# Study satisfaction among university students during the COVID-19 pandemic: Longitudinal development and personal-contextual predictors

**DOI:** 10.3389/fpsyg.2022.918367

**Published:** 2022-08-22

**Authors:** Christopher K. Gadosey, Carola Grunschel, Lena S. Kegel, Theresa Schnettler, Derya Turhan, Anne Scheunemann, Lisa Bäulke, Laura Thomas, Ulrike Buhlmann, Markus Dresel, Stefan Fries, Detlev Leutner, Joachim Wirth

**Affiliations:** ^1^Department of Educational Psychology, University of Münster, Münster, Germany; ^2^Department of Research on Learning and Instruction, Ruhr University Bochum, Bochum, Germany; ^3^Department of Psychology, University of Augsburg, Augsburg, Germany; ^4^Department of Assessment and Evaluation in Schools, University of Münster, Münster, Germany; ^5^Department of Clinical Psychology and Psychotherapy, University of Münster, Münster, Germany; ^6^Department of Psychology, Bielefeld University, Bielefeld, Germany; ^7^Department of Instructional Psychology, University of Duisburg-Essen, Essen, Germany

**Keywords:** COVID-19 pandemic, study satisfaction subdimensions, longitudinal development, motivational costs, loneliness, university students

## Abstract

The COVID-19 pandemic challenges the well-being and academic success of many students. Yet, little is known about students’ study satisfaction during the COVID-19 pandemic, a multilayered construct which accounts for students’ subjective cognitive well-being and academic success. Besides, previous studies on study satisfaction are mostly cross-sectional and hardly consider the distinct subdimensions of this construct. Therefore, our main goal in this study was to shed light on the understudied development of the subdimensions of study satisfaction (i.e., satisfaction with study content, conditions of studying, and coping with study-related stress) in two semesters amid the COVID-19 pandemic. Additionally, we examined how particular personal (i.e., gender, age, GPA, intrinsic motivation, motivational cost, and academic procrastination) and contextual (i.e., loneliness) factors are related to these subdimensions. We conducted two panel studies with convenience and purposeful samples of university students in Germany (*N*_*study*1_ = 837; *N*_*study*2_ = 719). Participants responded online to questions on each of the subdimensions of study satisfaction at the beginning, middle, and end of each semester but responded to measures of personal and contextual factors only at the beginning of each semester. In both studies, manifest growth curve models indicated a decrease in all subdimensions of study satisfaction as the semester progressed. Generally, gender (male) and intrinsic motivation were positive predictors but age (younger students), motivational cost, and loneliness were negative predictors of different subdimensions of study satisfaction – particularly satisfaction with study content. Overall, motivational costs and loneliness were the most consistent predictors of all subdimensions of study satisfaction across both studies. Our findings provide support for the understanding that study satisfaction could diminish in the face of challenging situations such as in this pandemic. The present study also highlights certain personal and contextual factors that relate to study satisfaction and calls for intensive research into the multidimensional construct of study satisfaction.

## Introduction

The COVID-19 pandemic has led to fundamental changes in everyday life and several aspects of teaching and learning in the higher education context. For instance, teaching has mainly been realized in digital environments while students have mostly studied on their own at home. The existing studies have often investigated how the pandemic has challenged students’ psychological health and well-being (e.g., Odriozola-González et al., 2020). Regarding students’ well-being in the COVID-19 pandemic, affective components of students’ subjective well-being such as mood (Diener et al., 2018) have been in the focus (Cao et al., 2020; Odriozola-González et al., 2020; Wang and Zhao, 2020), whereas the cognitive components of students’ subjective well-being like study satisfaction (Diener et al., 2018) are largely missing in the existing research. Yet, study satisfaction is not only a multifaceted construct but also it is considered a critical dimension of students’ academic success (York et al., 2015).

In the current COVID-19 pandemic, decreases in students’ study satisfaction are conceivable as students are exposed to several pandemic-related challenges (cf. Sahu, 2020). Yet, no studies exist on the trajectory of this multifaceted construct during this pandemic. Therefore, we investigated the development of the subdimensions of study satisfaction (i.e., satisfaction with study content, conditions of studying, and coping with study-related stress) among students in two longitudinal studies in Germany in the first (Study 1) and second (Study 2) online semesters of the COVID-19 pandemic. Furthermore, we examined how personal and contextual factors are associated with these subdimensions at the beginning and to changes over the semester.

The present study complements the limited understanding of the longitudinal development of subdimensions of study satisfaction and provides valuable insights into specific personal and contextual factors that might play important role in their development during the first and second COVID-19 semesters. Since reduced study satisfaction associates with impaired academic performance (Dhaqane and Afrah, 2016) and dropout from university (Fleischer et al., 2019), the present study could reveal important starting points for prevention and intervention at universities.

### Studying during the COVID-19 pandemic

Globally, students have faced concerns about their health and uncertain world events amid the COVID-19 pandemic (Baloran, 2020). This pandemic also posed several changes for studying at the university. Overall, the demand for self-regulation heightened than ever before (Aini et al., 2020). During the first (summer term 2020) and second (fall term 2020) COVID-19 semesters in Germany, teaching was realized in digital environments with asynchronous and synchronous courses (Guo, 2020). In the second semester, the quality of digital teaching might have improved based on first experiences in the first COVID-19 semester (Mishra et al., 2020; Saverino et al., 2021). Generally, students spent a lot of time in front of their computers and due to social contact restrictions, were often alone and had less interactions with others than before (Elmer et al., 2020). While some restrictions were eased in the 2020 summer semester and social interactions were partially possible in Germany, the restriction measures increased again in the following fall semester. In these challenging but slightly different situations in both semesters, students’ satisfaction with their studies may differ.

### Study satisfaction

Conceptual models (e.g., Kuh et al., 2006; York et al., 2015) consider study satisfaction as a core part and subjective indicator of academic success. It can also be described as the cognitive component of subjective well-being (Diener et al., 2002). Thus, study satisfaction lies at the intersection of academic success and subjective well-being. Essentially, study satisfaction captures students’ positive and negative cognitive evaluations of diverse aspects of their studies (Westermann and Heise, 2018). Schiefele and Jacob-Ebbinghaus (2006) posited that study satisfaction consists of three subdimensions, namely, satisfaction with “taught contents, conditions of studying, and coping with (study-related) stress” (p. 199). Specifically, satisfaction with taught contents represents students’ feelings of joy and satisfaction related to their chosen major or program. Satisfaction with conditions of studying refers to students’ experiences related to the university environment. Lastly, satisfaction with coping with study-related stress refers to students’ ability to manage academic stress in their personal lives (Wach et al., 2016).

Generally, empirical research on study satisfaction is limited and mainly cross-sectional (e.g., Grunschel et al., 2016; Bernholt et al., 2018). Due to scant longitudinal research, the theoretical understanding of its temporal development is limited. On a descriptive level, a few findings indicated a slight linear increase in overall study satisfaction over a period of 4 months (Ng and Ye, 2016) and 6 months (Allan et al., 2020) without considering the subdimensions. Contrary, Scheunemann et al. (2021) found a decrease in overall study satisfaction over one semester. Currently, during the COVID-19 pandemic, decreases in study satisfaction are conceivable as students are exposed to several pandemic-related challenges (Sahu, 2020). Besides, students have reported experiencing several challenges and dissatisfaction with digital education (the typical teaching format during COVID-19 pandemic) compared to face-to-face education (Bowers and Kumar, 2015; Maqableh and Alia, 2021).

Empirical studies—*prior to the COVID-19 pandemic*—unraveled a broad spectrum of personal and contextual determinants related to study satisfaction. For instance, personal determinants comprised sociodemographic characteristics (e.g., Nauta, 2007), academic motivation and personality characteristics (e.g., Wach et al., 2016), and dysfunctional study behavior like academic procrastination (e.g., Balkis and Duru, 2016). Contextual determinants encompassed social climate factors (e.g., Tompkins et al., 2016) and learning conditions (e.g., Bowers and Kumar, 2015; Yen et al., 2018). Mostly, these results stem from studies that used a composite score of study satisfaction rather than its subdimensions (exception: Wach et al., 2016). Yet, focusing on the subdimensions could throw light on the complex nature of study satisfaction (Schiefele and Jacob-Ebbinghaus, 2006) and help account for more differentiated relationships with its determinants.

In addition to the abovementioned determinants, particular factors triggered by the pandemic might also exist. For example, given the switch to digital learning environments and social contact restrictions due to COVID-19, students’ experiences of loneliness have increased (Elmer et al., 2020) and might have negatively influenced their study-related experiences like study satisfaction. To complement the limited existing research in this regard, the present study addressed how particular personal (i.e., sociodemographic characteristics, academic performance, study motivation, and academic procrastination) and contextual (i.e., loneliness) factors are related to the subdimensions of study satisfaction during the COVID-19 pandemic.

### Predictors of study satisfaction

#### Sociodemographic characteristics

In some studies, gender was not related to overall study satisfaction (Moghimi et al., 2021) or the subdimensions of study satisfaction (Schiefele and Jacob-Ebbinghaus, 2006). was non-significant. In other studies, women were more satisfied with study contents than men (Bernholt et al., 2018) and men were more satisfied with study conditions and coping with study-related stress than women (Wach et al., 2016). Additionally, students’ age either did not relate (Moghimi et al., 2021) or related negatively to study satisfaction (Kegel et al., 2021), suggesting that younger students reported higher study satisfaction.

#### Academic performance

So far, only a few findings exist on the relationship of study satisfaction with university grade point average (GPA)—a widely used indicator of academic performance. Generally, GPA is positively associated with overall study satisfaction (Meneghel et al., 2019; Moghimi et al., 2021). However, the differentiated examination of the subdimensions of study satisfaction showed that only satisfaction with study conditions related positively to GPA (Wach et al., 2016).

#### Student motivation

Students’ motivation emerged as a key predictor of study satisfaction in traditional (Bergey et al., 2018) and digital learning environments (Kim and Frick, 2011). We conceptualize students’ academic motivation in line with Situated Expectancy-Value Theory (SEVT; Eccles and Wigfield, 2020). The SEVT proposes that students choose, engage, persist, and achieve in a task if they consider themselves capable to perform well in it (expectancy for success) and care about it (subjective task value). Thereby, subjective task value subsumes a variety of positive (i.e., intrinsic value) and negative task appraisals (cost). Empirical evidence showed that amongst the positive value components, intrinsic value (also *interest value or intrinsic motivation*) positively related to satisfaction with study contents and study conditions (Wach et al., 2016).

Despite the unique role of cost in reflecting important barriers to successful learning, cost has been widely understudied in higher education (Flake et al., 2015; Beymer et al., 2020). Costs may include different aspects, for instance *effort* students must expend, an *emotional* burden, and the *loss of valuable alternative activities* (Eccles and Wigfield, 2020). First evidence showed that a composite score of cost negatively related to all subdimensions of study satisfaction, strongest for satisfaction with coping with study-related stress in traditional learning environments (Kryshko et al., 2022). Given that motivational costs were found to increase during the pandemic (Hilpert et al., 2021), a closer look at its relationship with study satisfaction will be insightful.

#### Academic procrastination

Procrastination is defined as “the voluntary delay of an intended and necessary and/or [personally] important activity, despite expecting potential negative consequences that outweigh the positive consequences of the delay” (Klingsieck, 2013, p. 26). Given that study-related intentions are not put into action, academic procrastination is considered as a self-regulation failure (Pychyl and Flett, 2012). Generally, academic procrastination is widespread among students (Klingsieck, 2013; Asio, 2020). Shuen et al. (2021) recently revealed that more than 40% of surveyed students reached high or moderate procrastination scores amid the pandemic.

In cross-sectional studies, academic procrastination is associated with low study satisfaction (e.g., Grunschel et al., 2016; Balkis and Duru, 2016). In a longitudinal study across one semester, Scheunemann et al. (2021) investigated the cross-lagged relations between academic procrastination and overall study satisfaction (beside students’ dropout intentions). At each measurement point, academic procrastination related negatively with study satisfaction. In the cross-lagged relations, high study satisfaction at the middle of the semester related to high procrastination at the end of the semester. The authors discussed this finding in terms of overconfidence in studies in the middle of the semester that was linked to more procrastination in the end. They related the contradictory results to the special characteristic of the sample that consisted mainly of freshmen students. Nevertheless, it is also possible that the continuous gap between intentions and actions can result in stress and negative emotions (Visser et al., 2018) which, then, can be linked to reduced study satisfaction in the long term (Grunschel et al., 2016).

#### Loneliness

Loneliness involves “feelings of isolation, feelings of disconnectedness, and feelings of not belonging” (Hughes et al., 2004, p. 657) and can be understood as an indicator of social integration. Because loneliness is subjective and contextual, neither being alone nor the mere amount of social relationships define loneliness (Hughes et al., 2004). Students are at a risk of feeling lonely due to their transition to college and strive to becoming independent (Diehl et al., 2018). During the COVID-19 pandemic, students experienced the conditions for social exchange as difficult (Marczuk et al., 2021) and felt lonelier (Elmer et al., 2020).

Different facets of social integration significantly relate to study satisfaction (Bernholt et al., 2018). Loneliness is accompanied by lack of social support (Kong and You, 2013), which can be linked to satisfaction with study conditions. Additionally, loneliness relates to high perceived stress in students (Stoliker and Lafreniere, 2015), which in turn can be linked to satisfaction with coping with study-related stress. However, studies examining the direction relation between loneliness and study s satisfaction and/or its subdimensions are still pending.

### The current research and hypotheses

The COVID-19 pandemic posed serious challenges to teaching and learning in the university context (Sahu, 2020). The situation also hampered students’ well-being (Cao et al., 2020). Surprisingly, study satisfaction, an interesting and multilayered construct which accounts for students’ subjective cognitive well-being (Diener et al., 2002) and academic success (Kuh et al., 2006), is largely left out in empirical studies during the COVID-19 pandemic. Besides, most existing research on study satisfaction is cross-sectional and only focused on the overall construct of study satisfaction. Yet, focusing on the subdimensions through longitudinal studies is useful for understanding the development of the subdimensions and also throws light on the role that personal and contextual factors play for each aspect of study satisfaction during the COVID-19 pandemic.

To extend existing research, we conducted two panel studies in the first and second COVID-19 semesters that included three measurement points each. At each measurement point (beginning, middle, and end of the lecture period of the semester), students reported their *satisfaction with study content* (S-Content), *satisfaction with study conditions* (S-Conditions), and *satisfaction with coping with study-related stress* (S-Coping). However, they responded to measures for the personal and contextual factors only at the beginning of the lecture period.

For both studies, we had the same objectives. The first objective was to examine whether the subdimensions of study satisfaction changed over the first (Study 1) and second COVID-19 semesters (Study 2; Research Question 1). Based on previous studies (e.g., Allan et al., 2020; Scheunemann et al., 2021), it is not conclusive whether the subdimensions of study satisfaction increase or decrease over the semester. However, the COVID-19 pandemic brought many challenges to students (Sahu, 2020), making them less likely to be satisfied with subdimensions of study satisfaction. We therefore expected that each dimension of study satisfaction—S-Content, S-Conditions, and S-Coping—would decrease from the beginning to the end of the lecture period.

The second objective was to examine how personal and contextual factors are related to the subdimensions of study satisfaction in the first and second COVID-19 semesters. Specifically, we investigated whether the factors predicted the subdimensions of study satisfaction at the beginning of the semester (Research Question 2) as well as changes in the subdimensions over the course of the semesters (Research Question 3). Due to lack of existing findings, we did not specify the nature of the relationships between our predictors and the subdimensions of study satisfaction at the beginning and changes over the semesters for Study 1 and Study 2. Instead, we stated hypotheses for the direction of the relationships between our predictors and each of the subdimensions of study satisfaction based on prior research.

Specifically, we expected S-Content to relate positively with intrinsic motivation (Wach et al., 2016) but negatively with motivational cost (Kryshko et al., 2022), and women to score higher on S-Content than men (Bernholt et al., 2018). Furthermore, we expected S-Conditions to be positively associated with GPA (Wach et al., 2016) and intrinsic motivation (Wach et al., 2016) but negatively associated with motivational cost (Kryshko et al., 2022). We also expected men to report higher S-Conditions (Wach et al., 2016). Lastly, we expected that S-Coping would be negatively related to motivational cost (Kryshko et al., 2022) and that men will report higher S-Coping (Wach et al., 2016).

Generally, based on previous findings with overall study satisfaction, we assumed possible negative relationships for age (Kegel et al., 2021), academic procrastination (e.g., Scheunemann et al., 2021), and loneliness (cf. Elmer et al., 2020) concerning the subdimensions of study satisfaction.

## Materials and methods

For both Study 1 and Study 2, we used the same research design, standardized measures, and statistical analyses. Only the samples differed between the two studies. Given our research interest in the development of university students’ study satisfaction in two COVID-19 semesters, we conducted panel studies. The longitudinal study design enabled us to assess our variables at multiple times and examine their trajectories and interrelations. Due to closure of university campuses during the COVID-19 pandemic, we carried out the recruitment of participants digitally. We opted for a convenience sample in Study 1 in order to recruit as many participants as possible from various German universities. In Study 2, we had a purposeful sample as we predetermined the sample as part of a larger research project (*refer Section “Procedure and sample”*). In the following, we describe the standardized measures that we used in our online surveys and explain the statistical analyses for both studies. We present the different samples for each study separately in their respective results sections.

### Variables and measures

We report those variables from the two longitudinal studies that are relevant to our research questions. As sociodemographic variables, students reported gender, age, and semesters studied. For the following described measures, reversed items were recoded so that higher values indicated higher expressions on the variables.

#### Study satisfaction

We assessed study satisfaction with the German version of the *study-satisfaction questionnaire* of Schiefele and Jacob-Ebbinghaus (2006). The scale consists of ten items and has three dimensions. Participants rated on a 6-point Likert scale (ranging from 1 = “*strongly agree”* to 6 = “*strongly disagree”*) to what extent they were satisfied with the *study contents* (e.g., “I really enjoy the subject of my studies”), *conditions of studying* (e.g., “I wish the study conditions at my university were better”), and *coping with study-related stress* (e.g., “I am not able to reconcile my study requirements with other personal obligations”). The overall study satisfaction scale and each of the subscales had satisfying reliability at each measurement point: overall scale (Study 1: ω = 0.83; Study 2: ω = 0.85); S-Content (Study 1: ω = 0.88; Study 2: ω = 0.87); S-Conditions (Study 1: ω = 0.81; Study 2: ω = 0.83), and S-Coping (Study 1: ω = 0.80; Study 2: ω = 0.84).

#### Academic performance

Participants reported their current GPA as a measure of their academic performance. The GPA ranges from 1.0 (*sufficient*) to 4.0 (*very good*).

#### Student motivation

We assessed two relevant aspects of student motivation, a positive value component (intrinsic value) and a negative value component (motivational cost). In Study 1, all items referred to studying in general, whereas in Study 2, the items targeted students’ major. We employed three items adapted to the higher education context (Schnettler et al., 2020a,b) to assess participants’ intrinsic value for their studies (e.g., “My major is fun to me”). For motivational cost, we adapted nine items from Schnettler et al. (2020a,b). We employed three items per each of the three cost facets: effort cost (e.g., “Studying my major is exhausting to me”), emotional cost (e.g., “My major is a real burden to me”), and opportunity cost (e.g., “I have to give up a lot to do well in my major”). Responses for all items were on a 6-point Likert scale, ranging from 1 = *strongly disagree* to 6 = *strongly agree.* For the analyses, we combined the cost items into a general cost score (cf. Beymer et al., 2020). This was supported by a higher order cost factor in confirmatory factor analyses^1^ (Study 1: χ^2^ = 135.97, df = 25, *p* < 0.001, CFI = 0.97, RMSEA [90% CI] = 0.073 [0.061, 0.085], SRMR = 0.039; Study 2: χ^2^ = 101.67, df = 24, *p* < 0.001, CFI = 0.97, RMSEA [90% CI] = 0.067 [0.054, 0.081], SRMR = 0.029). Reliabilities were good for intrinsic value (Study 1: ω = 0.88; Study 2: ω = 0.90) and motivational cost (Study 1: ω = 0.90; Study 2: ω = 0.92), respectively.

#### Academic procrastination

We measured academic procrastination with the German version of the Tuckman Procrastination Scale (TPS-d; Tuckman, 1991; Stöber, 1995) explicitly adapted to the academic context (Grunschel et al., 2013). The TPS-d consists of 16 items describing students’ delay of study-related tasks (e.g., “I needlessly delay the completion of work in my studies, even if they are important”). Responses were on a 5-point Likert scale (1 = *this is not at all true* to 5 = *this is very true*), and reliability was good (Study 1: ω = 0.94; Study 2: ω = 0.95) in the present study.

#### Loneliness

We assessed participants’ loneliness with the German version of the UCLA Loneliness Scale [Schützenberger, 2015; original version by Hughes et al. (2004)]. This three-item scale (e.g., “How much of the time do you feel isolated from others?”) has responses on a 3-point Likert scale (1 = *hardly ever*, 2 = *some of the time*, 3 = *often*). The scale had a satisfying internal consistency (Study 1: ω = 0.81; Study 2: ω = 0.85) in the present study.

### Statistical analyses

For both Study 1 and Study 2, we first evaluated the panel attrition, descriptive statistics, and bivariate correlations of all variables of interest using SPSS Version 27. For the panel attrition, we checked whether the attrition of participants in our study had associations with overall study satisfaction, age, gender, and GPA. Significant differences between the groups suggested systematic missingness (Enders, 2010)—which was the case (*refer Section “Procedure and sample”*). We addressed systematic missingness by applying multiple imputation procedure. The multiple imputation approach has been shown to reduce wastefulness of data and biased results compared to the use of complete cases (Asendorpf et al., 2014; van Ginkel et al., 2020). We followed the guidelines of Geiser (2021) and implemented multiple imputation with Mplus 8.7 software (Muthén and Muthén, 1998, 2017).

Subsequently, we examined the longitudinal development of each subdimension of study satisfaction over each semester. To first determine the nature of change in each subdimension, we compared intercept-only models (i.e., specifying only mean intercepts or initial levels) and linear unconditional first-order latent growth curve models (LGCM; i.e., specifying both mean intercepts and mean slopes or developmental trajectories) for each subdimension separately. While intercept-only models postulate no (linear) change over time, LGCMs include linear increase or decrease over time. We fixed the coefficients of the intercept growth factor at 1 and the time scores for the slope growth factor at 0, 1, and 2 (cf. Muthén and Muthén, 1998–2017). In the next step, we specified one bigger model that integrated the best-fitting models (intercept-only or linear LGCM) for each subdimension. This bigger model yielded insights into the longitudinal relationship of the subdimensions.

Lastly, with linear conditional first-order LGCMs, we examined the predictive role of our personal and contextual factors for the subdimensions. In this model, we estimated the mean intercepts (i.e., initial levels) and mean slopes (i.e., changes) of each subdimension of study satisfaction. We fixed the coefficients of the intercept growth factors at 1 and the time scores for the slope growth factors at 0, 1, and 2. We finally added the personal and contextual factors as predictors of the intercepts and slopes^2^.

We followed the recommendations of Weston et al. (2008) and assessed the goodness of fit of the unconditional and conditional LGCMs with several indicators, namely, χ^2^ test statistic, standardized root-mean square residual (SRMR), and comparative fit Index (CFI). Specifically, a model was deemed to have good fit when SRMR ≤ 0.08 and CFI values ≥ 0.95. Following Kenny et al. (2015), we did not report root-mean-square error of approximation (RMSEA) because RMSEA is discussed to be misleading in models having only a small number of degrees of freedom such as ours.

## Results of study 1

### Procedure and sample

Study 1 took place in Germany during the lecture period of the first COVID-19 semester (T1: end of April; T2: middle of June; T3: ending of July 2020). We obtained approval from the university’s ethics committee before data collection. We recruited students from various German universities by sending e-mails to persons incharge of media communications in these universities who in turn contacted the students. We also shared the invitation to the online study on university students’ social media platforms. Interested students followed the given link to participate in the online study. To obtain a diverse convenience sample of students, our only main inclusion criterion required students to be currently enrolled in a degree program in a Germany university. Students received detailed information about the study and data privacy issues. They declared their informed consent before participating.

At T1, the sample consisted of *N* = 860 (*n* = 687 female, *n* = 170 male, *n* = 3 diverse) students in various programs such as natural sciences, humanities, social sciences, law, engineering, teaching, and music. At T2, *n* = 597 students and at T3, *n* = 473 students participated. The attrition rate between T1 and T3 was 49.4%, reflecting the difficulty students had with participating in panel studies amid the pandemic challenges. Nonetheless, this rate is comparable to other longitudinal studies (refer Deng et al., 2013). We determined that first semester students (*n* = 23) were to be excluded from our analyses on the basis that they had no complete records of their current academic performance. Therefore, we considered a sample of *N* = 837 students (*n* = 671 females; *n* = 163 males and *n* = 3 diverse) for our data analysis (T1 = 837, T2 = 557 and T3 = 449). At T1, these participants’ mean age was 23.72 years (*SD* = 4.13) and were in their 4.10 (*SD* = 2.59) semester. Participants could either obtain course credit if they were psychology students in the researchers’ university or participate in a raffle for vouchers worth up to 100 Euros.

### Panel attrition

We tested whether differences existed among our participants with regard to overall score of study satisfaction, age, gender, and GPA. Given that study satisfaction was not normally distributed and our comparison groups differed greatly in size, we opted for the non-parametric Kruskal-Wallis test to determine whether differences in study satisfaction existed among participants who completed all three measurement points (*n* = 424, 51%), only two measurement points (*n* = 158, 19%), or only T1 (*n* = 255, 30%). The analyses revealed significant differences [*H*(2) = 6.70, *p* = 0.04] between the three participant groups. Pairwise comparisons with adjusted p-values showed that students who participated in all three measurement points differed significantly in study satisfaction from students who participated in only T1 (*p* = 0.04, *r* = 0.10). Further attrition analyses revealed that there were no significant age differences [*H*(2) = 0.21, *p* = 0.90] per the number of measurement points a person completed. Participants who completed all three measurement points had better GPA (*p* = 0.04, *r* = −0.47) than participants who completed only T1. In addition, relatively more female than male participants completed all three measurement points compared to completing only T1 [χ*^2^*(4) = 14.86, *p* < 0.01]. Thus, we had systematic attrition in this study. To handle missing data, we used multiple imputations with 50 imputations (refer Asendorpf et al., 2014). Our results are based on these imputed data.

### Descriptive statistics and bivariate correlations

Table 1 displays the descriptive statistics and bivariate correlations among our predictor variables and the subdimensions of study satisfaction across all three measurement points in Study 1. On an interindividual level, S-Content and S-Coping reduced from T1 to T2 but slightly increased from T2 to T3. In contrast, S-Conditions decreased from T1 to T2 and slightly decreased from T2 to T3. In addition, we found significant positive correlations among the subdimensions of study satisfaction across the measurement points (0.31 ≤ *r* ≤ 0.86). Furthermore, each of the subdimensions of study satisfaction correlated positively with intrinsic motivation (low to high) and negatively with cost (moderate to high), procrastination (low to moderate), and loneliness (low to moderate).

**TABLE 1:** Means, standard deviations, and correlations between sociodemographics, predictor variables and subdimensions of study satisfaction in study 1.

		*M*	*SD*	1	2	3	4	5	6	7	8	9	10	11	12	13	14	15
1	Gender*^a^*	–	–	–														
2	Age	23.72	4.13	0.11**	–													
3	GPA	2.80	0.60	−0.06	0.10**	—												
4	Intrinsic motivation	4.55	0.94	0.01	0.08*	0.20***	—											
5	Motivational cost	3.01	0.96	0.02	0.07	−0.20***	−0.49***	—										
6	Procrastination	2.63	0.82	0.08*	0.01	−0.32***	−0.38***	0.30***	—									
7	Loneliness	3.29	1.16	−0.10**	−0.06	−0.11**	−0.15***	0.30***	0.18***	—								
8	S-CONT1	4.46	1.00	−0.01	0.01	0.21***	0.78***	−0.49***	−0.37***	−0.21***	—							
9	S-CONT2	4.32	1.03	−0.04	0.01	0.19***	0.74***	−0.48***	−0.36***	−0.19***	0.81***	—						
10	S-CONT3	4.33	1.06	−0.03	−0.02	0.24***	0.74***	−0.45***	−0.32***	−0.17***	0.81***	0.86***	—					
11	S-COND1	3.64	1.17	0.05	−0.09**	0.12**	0.31***	−0.38***	−0.20***	−0.17***	0.39***	0.40***	0.36***	—				
12	S-COND2	3.52	1.13	0.09*	−0.05	0.07	0.28***	−0.35***	−0.14***	−0.19***	0.34***	0.42***	0.39***	0.72***	—			
13	S-COND3	3.50	1.19	0.05	−0.05	0.12**	0.24***	−0.34***	−0.12**	−0.19***	0.31***	0.35***	0.37***	0.69***	0.74***	—		
14	S-COP1	4.12	1.09	0.08*	−0.07*	0.17***	0.38***	−0.79***	−0.26***	−0.29***	0.41***	0.41***	0.39***	0.41***	0.37***	0.31***	—	
15	S-COP2	3.94	1.11	0.06	−0.04	0.17***	0.35***	−0.73***	−0.23***	−0.25***	0.37***	0.44***	0.44***	0.35***	0.42***	0.38***	0.75***	—
16	S-COP3	3.96	1.16	0.09*	−0.02	0.21***	0.36***	−0.73***	−0.23***	−0.28***	0.36***	0.43***	0.45***	0.38***	0.39***	0.45***	0.72***	0.80***

*N* = 837. GPA, grade point average; S-CONT, satisfaction with study content; S-COND, satisfaction with study conditions; S-COP, satisfaction with coping with study related stress.

^a^1 = females and 2 = males.

*p < 0.05, **p < 0.01, ***p < 0.001.

### Changes in study satisfaction subdimensions

Overall, the unconditional linear LGCM (specified to examine changes in intercepts and slopes) across all subdimensions appeared to show better fit than the intercept-only models (refer Table 2). Therefore, we integrated the unconditional linear LGCM for each subdimension of study satisfaction in the bigger model. This bigger model showed satisfactory fit indices (χ^2^ = 124.55, df = 18, CFI = 0.979, TLI = 0.958, SRMR = 0.025). Table 3 presents the unstandardized parameter estimates of these LGCMs. The intercepts indicated that students reported moderate-to-high levels in the study satisfaction dimensions, meaning that they started the semester more satisfied. The significant negative slopes pointed to slight decreases in all dimensions over time. Thus, our hypotheses that each of the subdimensions of study satisfaction would decrease over the semester were supported.

**TABLE 2:** Fit statistics for intercept-only models and linear unconditional first-order latent growth curve models.

	Study 1				Study 2			
Model	χ^2^	*df*	CFI	SRMR	χ^2^	*df*	CFI	SRMR
**S-content**								
Intercept only	76.81	4	0.952	0.156	26.56	4	0.977	0.034
Linear	19.33	1	0.988	0.018	2.37	1	0.999	0.007
**S-conditions**								
Intercept only	29.18	4	0.978	0.057	21.64	4	0.979	0.028
Linear	4.78	1	0.997	0.012	8.742	1	0.991	0.019
**S-coping**								
Intercept only	75.55	4	0.946	0.148	58.35	4	0.945	0.069
Linear	22.90	1	0.984	0.024	12.59	1	0.988	0.019

*N*_study1_ = 837, *N*_study2_ = 719 using the total sample data from T1 and the multiple imputation method. S-Content, satisfaction with study content; S-Conditions, satisfaction with study conditions; S-Coping, satisfaction with coping with study-related stress. χ^2^, Yuan–Bentler robust test statistic; *df*, degrees of freedom; CFI, comparative fit index; SRMR, standardized root mean square residual.

**TABLE 3:** Unstandardized parameter estimates for the unconditional LGCMs in study 1 and study 2.

		Intercept		Slope		Covariance
Model		Mean	Variance	Mean	Variance	
**S-content**						
	Study 1	4.31***	1.00***	−0.06***	0.02	0.07*
	Study 2	4.23***	0.94***	−0.06***	0.02	−0.03
**S-conditions**						
	Study 1	3.48***	1.04***	−0.07***	0.02	0.04
	Study 2	3.59***	1.01***	−0.06***	0.01	−0.01
**S-coping**						
	Study 1	3.93***	1.14***	−0.08***	0.05	0.12**
	Study 2	3.95***	1.17***	−0.10***	0.06*	−0.05

*N*_1_ = 837 and *N*_2_ = 719 using the total sample data from T1 and the multiple imputation method.

S-content, satisfaction with study content; S-conditions, satisfaction with study conditions; S-coping, satisfaction with coping with study-related stress.

*p < 0.05, **p < 0.01, ***p < 0.001.

The significant variances indicated that inter-individual (between-person) differences existed in the intercepts. However, no meaningful differences existed in the slopes of all dimensions. The covariances were significant in the case of S-Content and S-Coping, showing that students with initially high values in these dimensions also reported higher changes in these dimensions over time.

### Predictions of satisfaction subdimensions at T1 and change over time

The conditional LGCM assessed whether inter-individual differences in the intercepts (i.e., at T1) and slopes of the dimensions (changes based on T1, T2, and T3) possibly existed in terms of personal factors (gender, age, GPA, academic procrastination, intrinsic motivation, and motivational costs) and contextual factors (loneliness). The well-fitting conditional LGCM (χ2 = 182.87, df = 39, CFI = 0.979, TLI = 0.947, SRMR = 0.014) explained relatively large amounts of variance in the subdimensions of S-Content (61–76%) and S-Coping (61–78%) compared to the relatively small amount of variance explained in S-Conditions (19–25%). Table 4 summarizes all results. In the following, we only report those findings that were significant for each subdimension of study satisfaction^3^.

**TABLE 4:** Prediction of study satisfaction subdimensions at T1 and changes over time in study 1 (standardized coefficients).

Predictors	S-content		S-conditions		S-coping	
	Intercept T1	Slope	Intercept T1	Slope	Intercept T1	Slope
Gender*^a^*	−*0*.*03*	−*0*.*02*	0.10*	−*0*.*11*	0.10***	0.02
Age	−*0*.*04*	−*0*.*1*	−*0*.12*	0.25	−*0*.*03*	0.06
GPA	0.01	−*0*.21*	0.01	0.19	0.01	0.13
Intrinsic motivation	0.75***	0.09	0.20***	−*0*.*29*	−*0*.*03*	0.04
Motivational costs	−*0*.13***	0.04	−*0*.30***	0.03	−*0*.86***	0.05
Procrastination	−*0*.08**	0.18	−*0*.*05*	0.43	−*0*.*04*	0.08
Loneliness	−*0*.08**	0.07	−*0*.*08*	−*0*.*28*	−*0*.*04*	−*0*.*03*
*R* ^2^	0.76	0.09	0.25	0.25	0.78	0.04

*N* = 837 using the total sample data from T1 and the multiple imputation method. S-content, satisfaction with study content; S-Conditions, satisfaction with study conditions; S-Coping, satisfaction with coping with study-related stress.

^a^1 = females and 2 = males.

*p < 0.05, **p < 0.01, ***p < 0.001.

#### Satisfaction with study content

Intrinsic motivation is strongly positive but motivational cost weakly negative related to the intercept of S-Content. Thus, students who reported higher intrinsic motivation and lower motivational costs were more satisfied with study contents at T1 (i.e., beginning of the semester). Additionally, lower academic procrastination and lower loneliness were weakly associated with higher S-Content at T1. The slope of S-Content was only negatively related to GPA, meaning that greater change (decreases) of S-Content over time occurred among students with a lower GPA.

#### Satisfaction with study conditions

Intrinsic motivation is weakly positive but motivational costs moderately negative related to the intercept of S-Conditions. Thus, students who reported higher intrinsic motivation and lower motivational costs were more satisfied with study conditions at T1. In addition, gender (i.e., males) is weakly positive and age (i.e., younger students) is weakly negative related to S-Conditions at T1.

#### Satisfaction with coping with study-related stress

Motivational costs strongly negative and gender (i.e., males) is weakly positive related to the intercept of S-Coping. Thus, students with lower motivational costs and male students reported higher S-Coping at the beginning of the semester.

### Summary of results

In sum, the results indicated that, on average, students’ satisfaction with their study contents, conditions of studying, and coping with study-related stress was moderate to high at the beginning of the semester but seemingly decreased as the semester progressed. Furthermore, motivational cost was a negative predictor of all subdimensions of study satisfaction, whereas intrinsic motivation was a positive predictor of only S-Content and S-Conditions at the beginning of the semester. Also, academic procrastination and loneliness negatively predicted S-Content. The sociodemographic variables of age negatively predicted S-Conditions (higher for younger students) and gender positively predicted both S-Conditions and S-Coping (higher for male students). Apart from GPA which was a positive predictor of the slope of S-Content, none of our predictors related to the changes (slopes) in the subdimensions over the semester.

## Results of study 2

### Procedure and sample

Study 2 took place in Germany during the lecture periods of the second COVID-19 semester as part of a larger research project funded by the German Federal Ministry of Education and Research. This larger study spread across the period of 2018–2021 with 13 measurement points. For our study, we used data of measurement points T7 to T9 (T7: October/beginning of November 2020; T8: middle of December; T9: beginning of February 2021). The larger study aimed at examining the risk factors of student dropout intentions from a motivation-action-regulation perspective in selected academic majors with moderate (law, economics, 27%; Heublein, 2014) and high dropout rates in Germany (STEM disciplines, 39%; Heublein, 2014). We collected data mainly from three federal German universities with approval from the ethics committee. Students enrolled in one of the selected majors were eligible for participation in the study. We invited the participants to the online study by e-mails and informed them prior to their participation about the purpose of the study and data privacy issues.

We excluded from our analyses first semester students, students who did not enroll for the fall semester, and persons who did not give consent for data processing. At T7, *N* = 719 (*n* = 537 women; *n* = 180 men; *n* = 2 diverse and *n* = 1 unspecified) participated. On average, they were 23.04 (*SD* = 3.53) years old and in their 5.25 (*SD* = 2.04) semester. At T8, *n* = 594 students and at T9, *n* = 577 students participated. The attrition rate between T7 and T9, which was 24.1%—relatively better than Study 1—suggests that more students were committed to participate in this panel study. Participants could receive up to 20 Euros for their participation from T7 to T9, depending on their individual compliance rate. To facilitate understanding of our results, we designated T7, T8, and T9 to equal T1, T2, and T3, respectively, for Study 2.

### Panel attrition

Similar to Study 1, we tested for Study 2 whether differences existed among our participants with regard to overall score of study satisfaction, age, gender, and GPA. Again, we opted for the non-parametric Kruskal-Wallis test to determine whether differences existed among participants who completed all three measurement points (*n* = 546, 76%), only two measurement points (*n* = 78, 11%), or only T1 (*n* = 95, 13%). The analyses revealed significant differences [*H*(2) = 9.14, *p* = 0.01] between the three participant groups. Pairwise comparisons with adjusted *p*-values showed that students who participated in all three measurement points differed significantly in study satisfaction from students who participated in only T1 (*p* = 0.008, *r* = 0.12). Further attrition analyses revealed that there were no significant differences in age [*H*(2) = 2.14, *p* = 0.34], GPA [*H*(2) = 3.59, *p* = 0.17], or gender [χ*^2^*(1) = 0.98, *p* = 0.99] regarding the three participant groups. Due to the significant differences in study satisfaction between the participant groups, our data had systematic attrition. We therefore employed multiple imputations with 50 imputations to handle missing data (Asendorpf et al., 2014). Our results are based on these imputed data.

### Descriptive statistics and bivariate correlations

The descriptive statistics and bivariate correlations among our predictor variables and the subdimensions of study satisfaction across the three measurement points were similar in Study 2 as compared to Study 1 (refer Tables 1, 5). That is, on an interindividual level, each of the subdimensions of study satisfaction decreased from T1 to T2 and slightly decreased from T2 to T3. The estimated means of the subdimensions were slightly higher in Study 1 than in Study 2. There were significant positive correlations among the subdimensions of study satisfaction across the measurement points (0.25 ≤ *r* ≤ 0.85). Furthermore, each of the subdimensions of study satisfaction correlated positively with intrinsic motivation (low to high) and negatively with cost (moderate to high), procrastination (low to moderate), and loneliness (low to moderate).

**TABLE 5:** Means, standard deviations, and correlations between sociodemographics, predictor variables and subdimensions of study satisfaction in study 2.

		*M*	*SD*	1	2	3	4	5	6	7	8	9	10	11	12	13	14	15
1	Gender*^a^*	–	–	–														
2	Age	23.04	3.53	0.13**	–													
3	GPA	2.7	0.65	−*0*.*05*	0.05	–												
4	Intrinsic motivation	4.57	0.93	−*0*.*01*	−*0*.*02*	0.26***	–											
5	Motivational cost	3.27	1.00	0.00	0.06	−*0*.30***	−*0*.44***	–										
6	Procrastination	2.74	0.89	0.09*	0.07	−*0*.30***	−*0*.35***	0.33***	–									
7	Loneliness	3.39	1.31	−*0*.*04*	0.00	−*0*.*08*	−*0*.19***	0.33***	0.22***	–								
8	S-CONT1	4.24	1.05	0.01	−*0*.*01*	0.27***	0.78***	−*0*.54***	−*0*.39***	−*0*.28***	–							
9	S-CONT2	4.16	0.93	−*0*.*01*	0.00	0.29***	0.73***	−*0*.53***	−*0*.37***	−*0*.28***	0.85***	–						
10	S-CONT3	4.13	0.97	−*0*.*01*	0.00	0.22***	0.63***	−*0*.50***	−*0*.36***	−*0*.26***	0.79***	0.81***	–					
11	S-COND1	3.56	1.16	0.00	−*0*.11**	0.11**	0.20***	−*0*.38***	−*0*.21***	−*0*.26***	0.28***	0.27***	0.26***	–				
12	S-COND2	3.58	1.12	−*0*.*03*	−*0*.12**	0.06	0.22***	−*0*.35***	−*0*.18***	−*0*.23***	0.28***	0.32***	0.30***	0.76***	–			
13	S-COND3	3.43	1.18	−*0*.*06*	−*0*.*06*	0.10*	0.16***	−*0*.31***	−*0*.20***	−*0*.22***	0.25***	0.25***	0.30***	0.71***	0.72***	–		
14	S-COP1	3.96	1.16	0.06	−*0*.12**	0.23***	0.40***	−*0*.80***	−*0*.27***	−*0*.37***	0.50***	0.48***	0.45***	0.50***	0.44***	0.39***	–	
15	S-COP2	3.78	1.16	0.01	−*0*.10*	0.18***	0.36***	−*0*.75***	−*0*.24***	−*0*.37***	0.41***	0.46***	0.39***	0.43***	0.46***	0.37***	0.80***	–
16	S-COP3	3.77	1.17	0.02	−*0*.08*	0.24***	0.34***	−*0*.72***	−*0*.28***	−*0*.35***	0.42***	0.44***	0.49***	0.41***	0.40***	0.45***	0.76***	0.77***

*N* = 719. GPA, grade point average; S-CONT, satisfaction with study content; S-COND, satisfaction with study conditions; S-COP, satisfaction with coping with study related stress.

^a^1 = females and 2 = males.

*p < 0.05, **p < 0.01, ***p < 0.001.

### Changes in study satisfaction subdimensions

Overall, the unconditional and conditional linear LGCMs indicated similar results in Study 2 as compared to Study 1. Specifically, the unconditional LGCMs across all subdimensions appeared to show better fit than the intercept-only models (refer Table 2). As in Study 1, we thus integrated the unconditional linear LGCM for each subdimension of study satisfaction in the bigger model. This bigger model showed satisfactory fit indices (χ^2^ = 118.97, df = 18, CFI = 0.975, TLI = 0.950, SRMR = 0.018). Table 3 presents the unstandardized parameter estimates of these LGCMs. The intercepts indicated that students reported moderate-to-high levels in the study satisfaction dimensions, meaning that they started the semester more satisfied. The significant negative slopes pointed to slight decreases in all dimensions over time, supporting our hypotheses that each study satisfaction dimension would decrease as the semester progresses.

The significant variances indicated that inter-individual (between-person) differences existed in the intercepts. In addition, the slopes of S-Coping differed, meaning that changes in students’ satisfaction with coping with study-related stress varied. We identified no significant co-variances between the intercept and slope of any dimension of study satisfaction.

### Predictions of satisfaction subdimensions at T1 and change over time

Similar to Study 1, conditional LGCMs in Study 2 yielded satisfactory fit indices (χ^2^ = 162.79, df = 39, CFI = 0.979, TLI = 0.946, SRMR = 0.014) and similar inter-individual differences in the intercepts (at T_1_) and slopes of the study satisfaction dimensions (changes based on T1, T2, and T3). In addition, the conditional LGCMs explained relatively large amounts of variance in the subdimensions of S-Content (62–79%) and S-Coping (68–79%) compared to the relatively small amount of variance explained in S-Conditions (18–24%). Table 6 summarizes all results. In the following, we only report those findings that were significant for each subdimension of study satisfaction^4^.

**TABLE 6:** Prediction of study satisfaction subdimensions at T1 and changes over time in Study 2 (standardized coefficients).

	S-content		S-conditions		S-coping	
	Intercept T1	Slope	Intercept T1	Slope	Intercept T1	Slope
Gender*^a^*	0.02	−0.18	0.01	−0.40	0.06*	−0.12
Age	0.02	0.06	−0.12**	0.46	−0.08**	0.10
GPA	0.03	−0.08	−0.03	−0.01	−0.02	0.09
Intrinsic motivation	0.70***	−1.02	0.04	−0.02	0.07**	−0.13
Motivational costs	−0.23***	−0.11	−0.35***	0.35	−0.81***	0.14
Procrastination	−0.08**	−0.15	−0.07	−0.05	0.02	−0.11
Loneliness	−0.07**	−0.09	−0.16***	0.05	−0.13***	−0.05
*R* ^2^	0.79	0.42	0.24	0.38	0.79	0.08

*N* = 719 using the total sample data from T1 and the multiple imputation method.

S-Content, satisfaction with study content; S-conditions, satisfaction with study conditions; S-coping, satisfaction with coping with study-related stress.

^a^1 = females and 2 = males.

*p < 0.05, **p < 0.01, ***p < 0.001.

#### Satisfaction with study content

Intrinsic motivation is strongly positive whereas motivational costs, academic procrastination, and loneliness are weakly negative related to the intercept of S-Content. Thus, students who reported higher intrinsic motivation but lower motivational costs, academic procrastination, and loneliness, were more satisfied with study-related contents at the beginning of the semester.

#### Satisfaction with study conditions

Motivational costs moderately negative and loneliness is weakly negative related to the intercept of S-Conditions at T1. Thus, students with lower motivational costs and feelings of loneliness were more satisfied with the study conditions at T1. Further, younger students reported higher S-Conditions at T1.

#### Satisfaction with coping with study-related stress

Motivational costs strongly negative and loneliness is weakly negative related to the intercept of S-Coping at T1. Further, intrinsic motivation was weakly positively related to S-Coping at T1, meaning that students with higher intrinsic motivation were more satisfied with their S-Coping at T1. Additionally, gender (male) was positively and age (younger students) negatively associated with this dimension at T1, indicating that male and younger students were more satisfied with S-Coping.

### Summary of results

Summarizing, the results in Study 2 were similar to those in Study 1. That is, students’ satisfaction with their study contents, conditions of studying, and coping with study-related stress was moderate to high at the beginning of the semester but decreased as the semester advanced. Additionally, motivational costs and loneliness were negative predictors of all the subdimensions of study satisfaction at the beginning of the semester. Moreover, intrinsic motivation was a positive predictor of S-Content and S-Coping, whereas academic procrastination again was a negative predictor of S-Content. Additionally, gender was a positive predictor of S-Coping (higher for male students), whereas age was a negative predictor of both S-Conditions and S-Coping (higher for younger students). None of our predictors related to the changes (slopes) in the subdimensions over the semester.

## Discussion

The COVID-19 pandemic is a challenge to students’ well-being (Odriozola-González et al., 2020) and could affect their study satisfaction. Yet, there is lack of studies on the multifaceted nature of study satisfaction especially during this pandemic. We therefore examined study satisfaction in detail in the first and second COVID-19 semesters. Expanding traditional research on the overall construct of study satisfaction, we differentiated between satisfaction with study content, study conditions, and coping with study-related stress. First, as extension to existing cross-sectional studies, we aimed to longitudinally investigate the development of the subdimensions of study satisfaction in two COVID-19 semesters. Subsequently, we sought to investigate the relations of personal and contextual factors with these subdimensions at the beginning of the semester as well as changes over the semester.

### Changes in the dimensions of study satisfaction

Our results showed a significant decrease for all three subdimensions of study satisfaction over the course of both the summer semester 2020 and fall semester 2020/2021. The decreasing trend found in our study is comparable to Scheunemann et al. (2021) although the drop in the means of the subdimensions over time were generally more during the pandemic. Our finding supports the understanding that study satisfaction is a subjectively assessed experience which could be dependent on and shaped by situations like the COVID-19 pandemic. While Scheunemann et al. (2021) considered the overall scores of study satisfaction, our study provides a detailed account of students’ experience of each subdimension of study satisfaction during the pandemic. In future research even beyond the pandemic, focus on the three dimensions would be insightful.

### Relations of study satisfaction with personal and contextual factors

Generally, in Study 1, S-Content and S-Conditions seemed related to both personal and contextual factors (i.e., loneliness), whereas S-Coping was mainly related to personal factors. Regarding Study 2, both personal and contextual factors significantly predicted all three subdimensions of study satisfaction. Overall, the relations of personal and contextual factors with the three subdimensions of study satisfaction were partly similar across the two studies.

#### Satisfaction with study content

Overall, all of our main personal and contextual predictors were significant predictors of S-Content suggesting that our predictors are indeed relevant for the experience of S-Content. Intrinsic motivation was the strongest and most consistent predictor of S-Content at the beginning of the semester across the two studies. This finding is consistent with Wach et al. (2016) and suggests that when students are interested and find what they study to be fun, they are more likely to be satisfied with their major. Also, motivational cost consistently negatively predicted S-Content across the two studies in line with the findings of Kryshko et al. (2022). This finding suggests that as students’ motivational costs increase, they tend to be dissatisfied with the content of their studies.

Furthermore, academic procrastination and loneliness appeared to consistently negatively relate to S-Content in both studies. The finding for academic procrastination is in line with Scheunemann et al. (2021) who found a negative relationship between academic procrastination and overall study satisfaction at each measurement point. There could be a mechanism operating such that students with higher tendencies to procrastinate tend to experience stress and negative emotions (Grunschel et al., 2016) and in turn become dissatisfied with their studies. This could be the focus of future studies to throw more light on how academic procrastination relates to the subdimensions of study satisfaction. Moreover, the findings with loneliness are rather new since loneliness has so far not been investigated in relation to the subdimensions of study satisfaction. This finding is reasonable amid the pandemic in which students felt lonely (Elmer et al., 2020) and likely lacked the needed peer and teacher interactions that could facilitate learning of taught contents.

Also, the slope of S-Content was significantly weakly related to GPA, meaning that greater changes (decrease) in S-Content over time occurred among students with a lower GPA compared to students with higher GPA. This finding suggests that students with higher GPA may have a more positive perception about their majors which in turn is associated with their satisfaction with the content of their studies.

#### Satisfaction with study conditions

In terms of S-Conditions, motivational costs were a consistent negative predictor at both time points across the two studies. This finding is in line with Kryshko et al. (2022) and suggests that when students perceive more motivational cost, they turn to be less satisfied with the university environment. This is likely the case in the pandemic times, when student had to adapt to the relatively new virtual learning environment rather than the physical learning environments they were used to (Hilpert et al., 2021).

Also, intrinsic motivation positively predicted S-Conditions in Study 1 (but not Study 2) which is consistent with Wach et al. (2016). This finding suggests that enthusiastic students may see the changes or shift to virtual learning environments and the efforts of the universities during the pandemic in a positive light. The lack of significant prediction of intrinsic motivation of S-Conditions in Study 2 may be an indication of different experiences in the two semesters. Loneliness also negatively predicted satisfaction with study conditions in Study 2. Again, this is a novel finding but reasonable since the pre-COVID-19 context most likely facilitated social interactions among students, which in contrast was lacking in the COVID-19 learning context (Hu and Gutman, 2021).

Moreover, age appeared as a negative predictor of S-Conditions at T1 in both studies meaning that younger students experienced higher S-Conditions at the beginning of each COVID-19 semester. It might be the case that the younger students, who are also mostly in the lower semesters, have less experiences with the study conditions and may not feel highly frustrated at least at the beginning of the semester. Finally, males turned out to be more satisfied with the S-Conditions at the beginning of Study 1 but this was not replicated in Study 2.

Overall, there is a relatively small amount of variance accounted for in the subdimension of S-Conditions in our study. This may suggest other important predictors than the personal variables that we have in this present study. Considering the contextuality of this subscale, factors such as access to online libraries and students’ evaluations of the online teachings offered to them during the COVID-19 pandemic may have played roles. Future studies should extend contextual variables when investigating this subdimension of study satisfaction.

#### Satisfaction with coping with study-related stress

Motivational costs were the strongest and most consistent predictor of S-Coping in both studies, which is in line with Kryshko et al. (2022). This finding is not surprising since the COVID-19 pandemic posed heavy challenges to students (Sahu, 2020), they could easily have difficulties dealing with these stressors as they experienced more motivational cost (Hilpert et al., 2021).

Furthermore, loneliness negatively predicted S-Coping at T1 in Study 2 (but not in Study 1). While this finding is novel, it is also not surprising since the lack of social interactions (also social support) during the COVID-19 pandemic must have left or overwhelmed students to deal mostly alone with the academic challenges that emerged. Loneliness however did not significantly predict S-Coping at T1 of the first COVID-19 semester. Perhaps, students may have adapted their coping strategies, as social contact restrictions were eased in the latter part of the first COVID-19 (summer) semester. In contrast, the second COVID-19 semester in fall began with a return to heavy social contact restrictions leading to students experiencing intense levels of loneliness (Hu and Gutman, 2021). This pattern may have accounted for loneliness consistently negatively predicting S-Coping in Study 2.

Moreover, gender appeared to consistently predict S-Coping across both studies in line with Wach et al. (2016). Overall, male students reported higher S-Coping. On the one hand, this may be due to female students feeling more stressed on average (Salmela-Aro and Read, 2017). On the other hand, it may suggest that male students perhaps have a better way of dealing with study-related stress. Future studies could throw more light on gender differences in this subdimension of study satisfaction.

Also, younger students and students with higher intrinsic motivation tend to be more satisfied with S-Coping at the beginning of the second COVID-19 semester. Similar to the findings with S-Conditions, younger students may begin their studies with less frustrations and may be satisfied with their stress-coping mechanisms at the beginning of the semester.

### Theoretical and practical implications

Although study satisfaction is an important aspect of cognitive well-being (Diener et al., 2002) and academic success (York et al., 2015), empirical research on study satisfaction with focus on the subdimensions of study satisfaction is largely neglected in research. Our present findings together with existing studies (e.g., Wach et al., 2016; Kryshko et al., 2022) show that more studies are needed in this growing area of research to deepen the understanding of the complex and differentiated nature of study satisfaction.

In the present study, our four main predictors (i.e., intrinsic motivation, motivational costs, academic procrastination, and loneliness) were all significant predictors of S-Content which was not the case for S-Conditions and S-Coping. Generally, it may be the case that some personal or contextual factors are more relevant for determining one or the other subdimension of study satisfaction. Our findings suggest to develop a more precise theoretical model on personal and contextual determinants of study satisfaction subdimensions that researchers should test in future research.

In this study, motivational costs stand out as the most consistent predictor of all the three subdimensions of study satisfaction in both Study 1 and Study 2. The experience of motivational cost seems to be closely related to lower study satisfaction. With this finding, interventions that aim at helping students deal with costs (e.g., Rosenzweig et al., 2019) could extend their focus and include study satisfaction as a relevant outcome in their trainings and evaluations.

Also, loneliness stands out as a consistent and relevant predictor of all the three subdimensions of study satisfaction in Study 2. On one hand, our study is the first to associate loneliness with the subdimensions of study satisfaction and suggests loneliness as a relevant determinant of study satisfaction especially in the context of the pandemic. One the other hand, our findings underline that working in groups and exchanges with peers in both physical and virtual forms seem to be important for study satisfaction in particular and well-being in general (refer Ullah and Wilson, 2007). Accordingly, contact among students in on-and-off-campus should be actively promoted by the university (refer Tinto, 1993).

Moreover, our findings concerning gender and age suggest that demographic variables could also play critical role in determining the satisfaction of university students. More socio-demographic variables could be examined to inform a comprehensive framework for the determinants of the subdimensions of study satisfaction. These findings could also be informative for counseling services to keep an eye on specific groups of students.

Also, there were hardly any relations of the predictors with the change in the dimensions of study satisfaction (slopes) except GPA which predicted the slope in S-Content. Future studies could examine how changes in the predictors relate to changes in the dimensions of study satisfaction.

Lastly, we found it surprising that academic procrastination hardly predicted the subdimensions of study satisfaction in the context of COVID-19 pandemic where self-regulation learning was key. Our study focused on procrastination tendencies of students’ (trait) and not procrastination behavior while learning (cf. Wieland et al., 2018). Possibly, behavior but not tendency is a better predictor of the subdimensions of study satisfaction. Future studies could use experience sampling methods to give insight into these possible relationships.

### Limitations and suggestions for future research

The present research has also some limitations. First, the studies were conducted with self-report measures. Although this is a frequent point of criticism in higher education research, we resorted to this approach because the central variable of our study, study satisfaction, is a subjective evaluation.

Second, due to convergence issues, the three subscales of study satisfaction were not modeled as latent constructs in the growth curve model. The complexity of the model with several predictors would have needed even greater sample size than we had. Accordingly, it was not possible to inspect measurement invariance. Overall, the higher education context needs more panel studies with more students.

Third, we performed the analyses using data partly from a convenience sample (Study 1) which displays disadvantaged generalizability relative to probability samples. In educational sciences, the usage of convenience samples is pervasive, given the cost-prohibitive nature of probability samples. However, we tried to minimize biased effects through a broad and easily accessible recruitment approach. Our efforts resulted in diverse samples. For instance, our data include multiple academic majors and students of different semesters.

Fourth, we resorted to multiple imputations to deal with missing data and to reduce bias in our results because we recorded substantial dropout over time leading to systematic attrition. In both samples, students who were more dissatisfied seem to have left the study. In Study 1 specifically, we found lower-performing (low GPA) and male students (who were already underrepresented in the sample) to drop out disproportionally. This is consistent with reasons for university student dropout (refer Heublein, 2014) indicating that dissatisfied and low-performing students leave higher education institutions more frequently without obtaining a diploma. Participation might thus have ended prematurely due to student dropout which was not recorded. Although such variance constraints typically increase the ß-error, we still found meaningful effects. Yet, we are careful with the interpretation of our results and the extent of generalizing our findings because the use of multiple imputation also has its limitations even though it is preferred over the use of, for example, complete cases (Asendorpf et al., 2014).

Lastly, we did not conduct Study 2 with the same sample as Study 1. Accordingly, the present results can only be partially compared for the purpose of gaining insights into the experiences of students during the heights of the COVID-19 pandemic. Both samples are heterogeneous, and this limits the extent of comparison. Future panel studies could follow the same samples to allow more comparisons between semesters and different study conditions. In addition, future studies could use the person-centered approach to investigate possible profiles of study satisfaction among university students in and out of challenging situations such as this pandemic.

### Conclusion

The present study provides detailed insights into the subdimensions of study satisfaction during the COVID-19 pandemic. Our study showed that each subdimension decreased across COVID-19 semesters. Also, important personal and contextual factors such as motivational cost, loneliness, intrinsic motivation as well as age and gender served to explain the distinct facets of study satisfaction – particularly satisfaction with content. Our findings are useful for tailoring interventions to assist students deal with their subjective well-being during hardships such as the COVID-19 pandemic. To conclude, we recommend more research into the subdimensions of study satisfaction to deepen the understanding of this interesting, dynamic, and multilayered construct.

## Data availability statement

The raw data supporting the conclusions of this article will be made available by the authors, without undue reservation, to any qualified researcher.

## Ethics statement

The studies involving human participants were reviewed and approved by Ethical Committee of the Department of Psychology, Bielefeld University and the Ethical Committee of the Institute of Psychology, University of Münster. Written informed consent for participation was not required for this study in accordance with the national legislation and the institutional requirements.

## Author contributions

CKG, CG, LK, TS, and DT contributed to conception and design of the study. TS, DT, AS, LT, and LB collected and organized the database. DT and TS handled the data analyses. CG, UB, MD, SF, DL, and JW supervised the study. CKG, CG, LK, TS, and DT wrote sections of the manuscript. All authors contributed ideas to prepare the manuscript, read and approved the final version of the manuscript for submission.

## Footnotes

1 When we computed the CFA in Study 1 without further constraints, a negative residual variance for effort cost was estimated. It was then fixed to 0. This resulting model did not lead to any further estimation problems (refer Beymer et al., 2020).

2 Additionally, we specified a second LGCM to examine the predictive role of our predictors for the end of semester (T3) experience of the subdimensions and to provide insight into the stability of the predictions. Here, we fixed the coefficients of the intercept growth factor at 1 and the time scores for the slope growth factors at, –2, –1, and 0. Thus, the means for the intercept in this model represented status at T3. Changes in time scores do not result in changes in the slopes, but in covariances (cf. Duncan and Duncan, 2009). The results of this analysis can be found in Supplementary Material.

3 In Supplementary Table 1, we additionally provide insights to what extend these personal and contextual factors predicted the intercepts at T3 (i.e., values at the end of the semester) of each study satisfaction subdimension.

4 In Supplementary Table 2, we additionally provide insights into the extent to which the personal and contextual factors predicted the intercepts at T3 (i.e., values at the end of the semester) of each study satisfaction subdimension.
