# Supplementary material for: Study satisfaction among university students during the COVID-19 pandemic: Longitudinal development and personal-contextual predictors
Source: Front Psychol. 2022 Aug 22;13:918367. doi: 10.3389/fpsyg.2022.918367 (PMC9441812; doi:10.3389/fpsyg.2022.918367)
Supplement: Supplementary file 1 [file Data_Sheet_1.docx]

| **Supplemental Table 1 \|** Prediction of Study Satisfaction Subdimensions at T3, and Changes over Time in Study 1 (Standardized Coefficients). | | | | | | | | | | | |  |
| --- | --- | --- | --- | --- | --- | --- | --- | --- | --- | --- | --- | --- |
| Predictors | S-Content | | |  | S-Conditions | | |  | S-Coping | | |  |
|  |  | Intercept T3 | Slope |  |  | Intercept T3 | Slope |  |  | Intercept T3 | Slope |  |
| Gender^a^ |  | -.03 | -.02 |  |  | .07 | -.11 |  |  | .10** | .02 |  |
| Age |  | -.07* | -.10 |  |  | -.06 | .25 |  |  | -.01 | .06 |  |
| GPA |  | .08* | -.21* |  |  | .05 | .19 |  |  | .07 | .13 |  |
| Intrinsic motivation |  | .70*** | .09 |  |  | .13* | -.29 |  |  | -.01 | .04 |  |
| Motivational costs |  | -.10** | .04 |  |  | -.28*** | .03 |  |  | -.74*** | .05 |  |
| Procrastination |  | -.01 | .18 |  |  | .04 | .43 |  |  | .18 | .08 |  |
| Loneliness |  | -.04 | .07 |  |  | -.12* | -.28 |  |  | -.05 | -.03 |  |
| R^2^ |  | .61 | .09 |  |  | .19 | .25 |  |  | .61 | .04 |  |
| *Note*.  *N* = 837 using the total sample data from T1 and the multiple imputation method. S-Content = Satisfaction with study content; S-Conditions = Satisfaction with study conditions; S-Coping= Satisfaction with coping with study-related stress. ^a^1 = females and 2 = males. **p* < .05. ***p* < .01. ****p* < .001. | | | | | | | | | | | |  |

| **Supplemental Table 2 \|** Prediction of Study Satisfaction Subdimensions at T3, and Changes over Time in Study 2 (Standardized Coefficients). | | | | | | | | | | | | | | | |  |
| --- | --- | --- | --- | --- | --- | --- | --- | --- | --- | --- | --- | --- | --- | --- | --- | --- |
|  | S-Content | | |  | | | S-Conditions | | |  | | | S-Coping | | |  |
|  |  | Intercept T3 | Slope | |  |  | | Intercept T3 | Slope | |  |  | | Intercept T3 | Slope |  |
| Gender^a^ |  | -.01 | -.18 | |  |  | | -.06 | -.40 | |  |  | | .02 | -.12 |  |
| Age |  | .03 | .06 | |  |  | | -.06 | .46 | |  |  | | -.05 | .10 |  |
| GPA |  | .02 | -.08 | |  |  | | -.03 | -.01 | |  |  | | .01 | .09 |  |
| Intrinsic motivation |  | .56*** | -1.02 | |  |  | | .04 | -.02 | |  |  | | .02 | -.13 |  |
| Motivational costs |  | -.25*** | -.11 | |  |  | | -.28*** | .35 | |  |  | | -.75*** | .14 |  |
| Procrastination |  | -.10** | -.15 | |  |  | | -.08 | -.05 | |  |  | | -.02 | -.11 |  |
| Loneliness |  | -.09** | -.09 | |  |  | | -.15** | .05 | |  |  | | -.15** | -.05 |  |
| R^2^ |  | .62 | .42 | |  |  | | .18 | .38 | |  |  | | .68 | .08 |  |
| *Note*.  *N* = 719 using the total sample data from T1 and the multiple imputation method. S-Content = Satisfaction with study content; S-Conditions = Satisfaction with study conditions; S-Coping= Satisfaction with coping with study-related stress. ^a^1 = females and 2 = males. **p* < .05. ***p* < .01. ****p* < .001. | | | | | | | | | | | | | | | |  |
